# Adaptive aberration correction using an electrowetting array

**DOI:** 10.1063/5.0133473

**Published:** 2023-02-21

**Authors:** Mo Zohrabi, Wei Yang Lim, Samuel Gilinsky, Victor M. Bright, Juliet T. Gopinath

**Affiliations:** 1 Department of Electrical, Computer and Energy Engineering, University of Colorado, Boulder, Colorado 80309, USA; 2 Department of Mechanical Engineering, University of Colorado, Boulder, Colorado 80309, USA; 3 Department of Physics, University of Colorado, Boulder, Colorado 80309, USA; 4 Materials Science and Engineering Program, University of Colorado, Boulder, Colorado 80309, USA

## Abstract

We demonstrate a method that permits wavefront aberration correction using an array of electrowetting prisms. A fixed high fill factor microlens array followed by a lower fill factor adaptive electrowetting prism array is used to correct wavefront aberration. The design and simulation of such aberration correction mechanism is described. Our results show significant improvement to the Strehl ratio by using our aberration correction scheme which results in diffraction limited performance. Compactness and effectiveness of our design can be implemented in many applications that require aberration correction, such as microscopy and consumer electronics.

Wavefront aberrations stem from refractive index mismatch of the mediums, off-axis transmission through the optical components, and medium inhomogeneity. They can be very detrimental in an optical system^1–3^ and can lead to a degradation of the point-spread function (PSF) resulting in low spatial resolution for an imaging system. Correcting the wavefront aberration is critical in many applications, such as astronomy and remote sensing,^4–10^ microscopy,^11–25^ and imaging.^26–28^ Adaptive systems have been implemented as efficient tools for correcting wavefront aberrations.^6,29–31^ A typical adaptive optical system is composed of compensation and measurement sections to correct aberration. A reconfigurable optical element like a deformable membrane mirror^32–37^ or a spatial light modulator^14,38,39^ modulates the wavefront to correct the aberration. Conventionally, the aberrations in the system are characterized by using a wavefront sensor, such as a Shack–Hartmann sensor.^40^ Recently, a wide variety of liquid-based optical devices have been demonstrated as adaptive optical elements to enhance optical performance,^41^ including electrowetting lenses and prisms,^42–58^ liquid crystals,^59–62^ pressure-driven elastic membranes,^63,64^ and other optofluidic devices.^65–68^

Electrowetting enables electrical tuning of the liquid droplet contact angle on a solid surface.^69^ The applied electric field decreases the interfacial energy between the liquid droplet and solid substrate, which results in “a decrease” in the droplet's contact angle and spreading out on the surface. Modern electrowetting devices consist of a two liquid system (polar and non-polar liquids) encapsulated in a cylindrical geometry.^42–51^ Miniature arrays based on electrowetting technology are compact, consume low power (*μ*W), enable kHz speeds of operation, and are transmissive. At the microscale size, electrowetting operation is solely dominated by a liquid's interfacial tension and the response time scales with the device diameters.^43,51,52,55^ Electrowetting arrays can be fabricated using standard two-dimensional lithography techniques to produce a 50–500 *μ*m diameter lenslet. Recently, aberration correction based on mm-size multi-electrode electrowetting devices has been demonstrated.^70,71^ In this work, we have simulated wavefront correction based on an array of electrowetting prisms. A 2D electrowetting prism consists of four or more electrodes around the perimeter of a cylindrical tube that can be individually controlled.^48,49,71^ These devices are capable of generating a tilted liquid–liquid interface as well as modifying the liquid–liquid curvature.

Here, we present simulations of a compact wavefront correction scheme based on a fixed microlens array followed by an electrowetting prism array. The fixed microlens and the electrowetting prism arrays are simulated in Zemax using a multi-configuration approach in a hexagonal pattern. To correct a given input aberration, the curvature and tilt of each prism element are varied and the optical performance of the system is evaluated by focusing the outgoing beam using an ideal lens. We demonstrate the ability to correct various aberrations with amplitudes ranging ±1 *μ*m by using an electrowetting prism array. The results of correcting three different input aberration examples (including astigmatism, coma, trefoil, and higher order aberration terms) are discussed and show large improvement in the point-spread function and the Strehl ratio, compared with the uncorrected cases.

The geometric configuration used in our simulation is based on an electrowetting “prism/lens” array fabricated using standard microfabrication techniques.^51,72–80^ The aberration correction scheme based on an electrowetting prism array is depicted in Fig. 1. In our simulation, the prism array is constructed in cylindrical tubes with a radius of 150 *μ*m and a pitch of 350 *μ*m, leaving a gap of 50 *μ*m between each electrowetting element as shown in Fig. 1(b). A tunable electrowetting prism array is placed after a fixed high fill factor microlens array (effective focal length 115 mm) and the aberrated wavefront is imaged onto the electrowetting prism array. The electrowetting prism array is simulated in a hexagonal grid with a fill factor 66.6%, while the fixed microlens array in a hexagonal closed-pack array has a fill factor of 90.7%. Designing an electrowetting prism array with lower fill factor reduces many of the fabrication difficulties for “the electrical connection” of each individual element. The fill factor of our design is restricted by the fixed microlens array (90.7%). Recently, a high-fill factor microlens array of 100% has been demonstrated using various fabrication methods.^81–85^ It is important to note that having a high fill factor electrowetting array is not necessary in our design. By placing the electrowetting lens array at the focal plane of the fixed microlens array [Fig. 1(a)], the effective fill factor is large by using a high fill factor fixed microlens array.

**FIG. 1.**
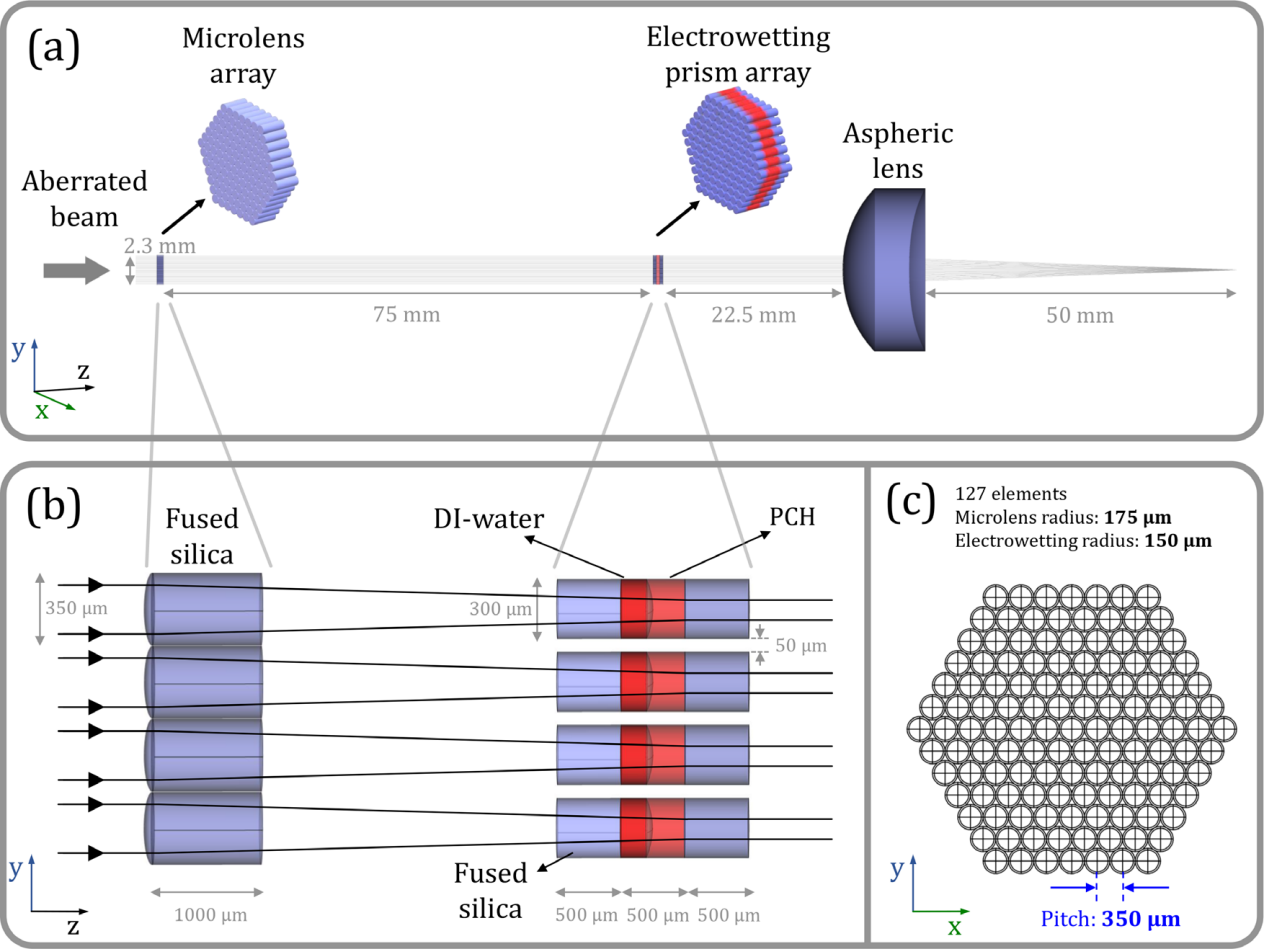
(a) Schematic of the aberration correction principle using a tunable electrowetting liquid prism array, a fixed microlens array, and an aspheric lens. The aspheric lens is used to evaluate the point-spread function in the imaging plane. This lens is designed to be aberration free in our system. (b) The microlens array has a radius of 175 *μ*m followed by an electrowetting prism with a radius of 150 *μ*m filled with de-ionized water and 1-phenyl-1-cyclohexene (PCH). (c) Cross-sectional view of the design with the microlens array and electrowetting prism overlayed. The fixed microlens array has a high fill factor of 90.7%, while the electrowetting prism array has a gap of 50 *μ*m between each element with a lower fill factor of 66.6%.

Figure 1(c) is the cross-sectional view of the system (xy-plane) that overlays the 127 microlens array with the electrowetting prism in a hexagonal pattern. The design has a pitch of 350 *μ*m and each prism element is filled with two immiscible liquids that are density matched, de-ionized (DI) water and 1-phenyl-1-cyclohexene (PCH), with a high refractive index contrast of Δn = 0.23. The initial contact angle of the liquid–liquid interface is altered by applying an electric field. Due to the electrowetting effect, this results in controlling the individual liquid–liquid curvature as well as the tilt in the x and y directions which is ideal for wavefront aberration correction. The aspherical lens shown in Fig. 1(a) is modeled as an asphere with no aberration for input beam diameters of ∼2.1 mm, as used in the simulation of microlens array and electrowetting prism. The aspheric lens is primarily used to generate an intensity distribution in the imaging plane, namely, point-spread function (PSF). Finally, the Strehl ratio of the optical setup (one measure of optical image quality) is evaluated and used as a feedback to the optimization algorithm for aberration correction.

To achieve the wavefront correction based on the principle described earlier, we simulated the optical system using sequential ray tracing in Zemax OpticStudio. We used the application programming interface (ZOS-API) in Zemax to interface with the Matlab environment in order to set up the simulation using 127 configurations corresponding to each array element, as shown in Fig. 1(a). Ideally, the liquid–liquid interface of each microlens element can be evaluated using the Young–Laplace equation or solving the Navier–Stokes equation.^70,86–89^ However, due to the large computation time required to calculate the liquid–liquid interface, we have used Zernike polynomials (specifically tip/tilt and defocus terms) to represent the liquid–liquid interface of each electrowetting microlens element. Our previous work showed that Zernike polynomials can be used as a least-square fit to represent the simulated liquid–liquid interface under any given voltage.^70^ This method speeds up the optimization process significantly despite having 3 × 127 coefficients. To correct the wavefront aberration, the curvature and tip/tilt of each liquid prism is optimized using a custom merit function. It is worth noting that by adjusting the curvature and tilt of the liquid–liquid interface, the volume of the two liquids is modified. We have added volume consistency to each microlens element as a correction for the Zernike fit method that we have chosen. For instance, when the liquid interface is defocused (or tilted) to compensate for the input aberration, the top and bottom liquid volume is altered. To correct this, we ensure that the volume consistency is applied to each microlens element to replicate the results from the 3D computational fluid dynamics simulation. To correct for the volume variations, the merit function evaluates the volume of each liquid section and forces them to be equal to the initial value using “VOLU” and “EQUA” operands. The center thickness value (“CTVA” operand) is calculated and the lens editor is adjusted for the given liquid–liquid interface curvature and tilt. We utilized a more efficient optimization process in which the optical path length for each configuration is evaluated using the “PLEN” operator and we ensured that the optical path lengths are identical between different configurations, resulting in an aberration-free system. In addition, the imaging efficiency of our design is evaluated using “IMAE” operands. Interfacing the Zemax API and Matlab is imperative, as the merit function is over 1400 lines of code. To correct the wavefront aberration, the curvature and tip/tilt (three variables) of each liquid prism array are optimized. In addition, we need to adjust the position of the liquid–liquid interface to ensure volume consistency resulting in four variables for each configuration (508 variables). We examined our design using basic aberrations based on Zernike polynomials.^90,91^ These polynomials are orthonormal over circular pupils and have widely been implemented in atmospheric and water turbulence compensation,^92–94^ in vision for ocular aberration measurements,^95,96^ in microscopy,^16,22,24,97,98^ and in optical metrology.^99–101^ The input aberration in our design is composed of a set of Zernike polynomimals—such as astigmatism (*Z*_5_, *Z*_6_), coma (*Z*_7_, *Z*_8_), defocus (*Z*_4_), spherical (*Z*_11_), and a combination of random aberrations (mix of *Z*_4_ to *Z*_12_).

We have studied three different cases containing different input aberrations, with peak-to-valley (PV) amplitudes ranging from ±0.24 to ±0.73 *μ*m at 632.8 nm. The aberration amplitudes are based on the reported values in various studies.^13,16,22–24,102–105^ For instance, McLellan *et al.* investigated the relation between age and vision quality of the eye and their measurements yield in Zernike coefficients amplitude of ±0.5 *μ*m.^102^ Zheng *et al.* studied multiphoton structured illumination microscopy using deformable mirrors and found out the aberration amplitudes of ranging from −0.1 to 0.4 *μ*m in a polyacrylamide gel.^104^ The initial contact angle of the electrowetting prism array was chosen to be 90°, translating into a flat liquid–liquid interface. The aspheric lens surfaces (even asphere surfaces) and its focal length are optimized to generate an aberration-free setup with a Strehl ratio ∼0.98 at the imaging plane. These parameters are then fixed and act as the initial condition for the simulation. The electrowetting prism array curvature and their tip/tilt angle are then optimized for various input aberrations through the system. To show the improvement, the PSF and Strehl ratio are evaluated. Finally, we have estimated the maximum angle of a wavefront that can be imaged on the aperture electrowetting prism and evaluated the maximum correction for a single prism element to be 0.6*λ*. The dynamic range of the system, assuming ten prism array elements in one direction, is ∼6*λ*. This is based on only wavefront tilt aberration and this limit breaks down for more complex wavefront aberrations that exhibit a steeper local slope.

Figure 2 shows the point-spread function (PSF) for three input astigmatism aberrations (*Z*_5_) with amplitudes of (a) ±0.24, (b) ±0.49, and (c) ±0.73 *μ*m in the left column. The Strehl ratios of these aberrated wavefronts are (a) 0.22, (b) 0.05, and (c) 0.025, respectively. We have optimized the 508 variables of our electrowetting prism array corresponding to curvature, tip, tilt, and the position of the liquid–liquid interface to ensure similar volumes for the top and bottom liquids. The corrected wavefront is shown in Figs. 2(d)–2(f) as PSF in the imaging plane. All of the input aberrations are corrected with the Strehl ratio of 0.95 satisfying the Maréchal criterion, in which the Strehl ratio above 0.80 in an optical system is considered to be diffraction limited.^3,106^

**FIG. 2.**
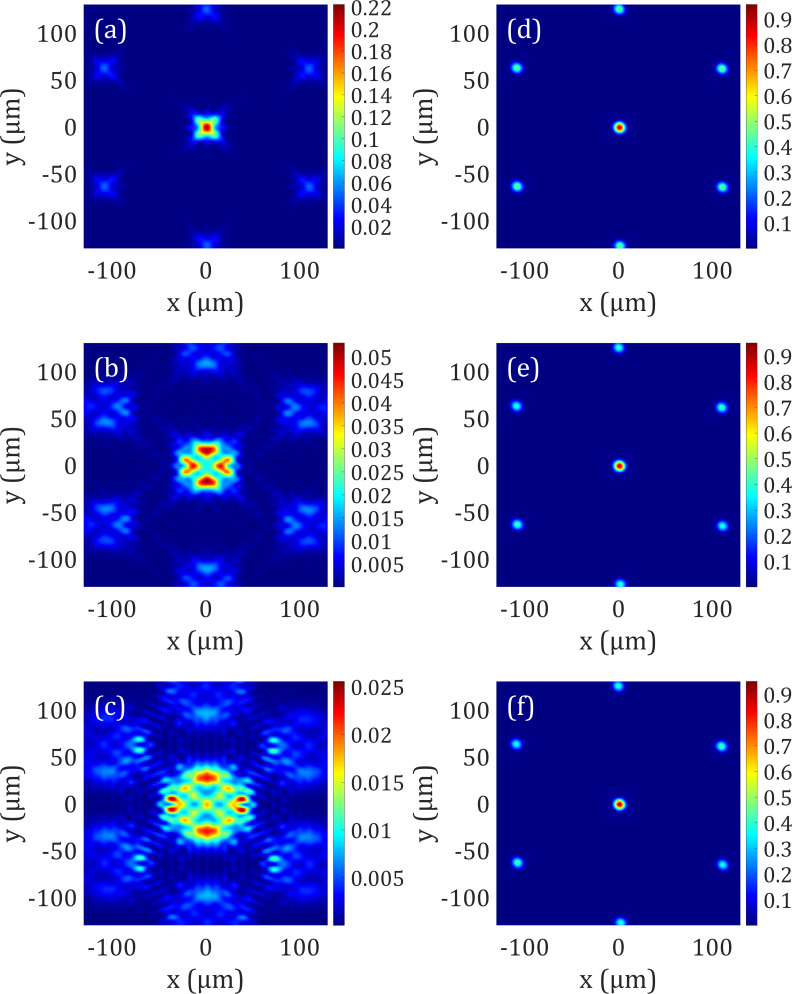
PSF, point spread function. Astigmatism aberrations (Zernike coefficient, *Z*_5_) are used as an input for our optical design with peak-to-valley (PV) amplitudes of (a) ±0.24, (b) ±0.49, and (c) ±0.73 *μ*m. (a)–(c) The corresponding PSF is plotted after focusing through an aspheric lens, with a Strehl ratio of (a) 0.22, (b) 0.05, and (c) 0.025. (d)–(f) The corresponding PSF is plotted after focusing through an aspheric lens and optimizing the liquid–liquid interface of the electrowetting prism array with a corrected Strehl ratio of (a) 0.958, (b) 0.951, and (c) 0.951. The Strehl ratio shows a significant improvement of the PSF satisfying the Maréchal criterion.

In the second case, we demonstrate the capability of our technique to correct coma aberration. The input coma aberration (Zernike coefficient, *Z*_7_) peak-to-valley amplitudes are the same as the astigmatism case. Figure 3 shows the PSF at the imaging plane for these aberrated wavefronts with the Strehl ratio of (a) 0.27, (b) 0.11, and (c) 0.08, respectively. The corrected wavefronts are graphed in Figs. 3(d)–3(f) with a corrected Strehl ratio of ∼0.90 satisfying the Maréchal criterion of the Strehl ratio >0.8.

**FIG. 3.**
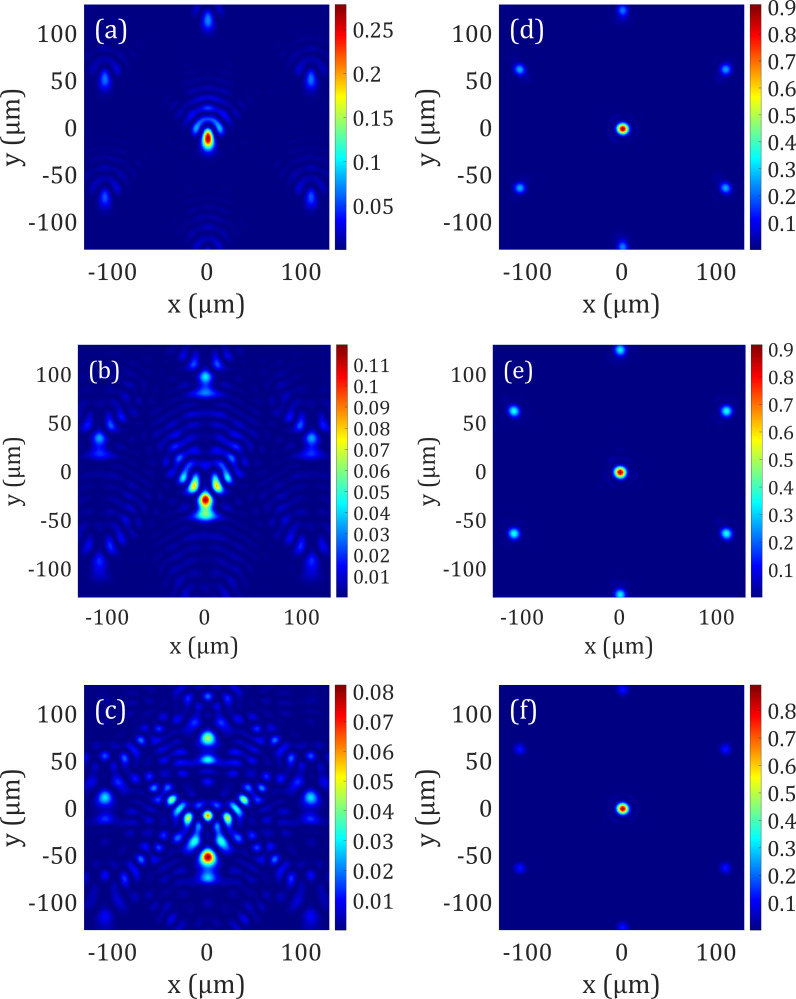
PSF, point spread function. Coma aberrations (Zernike coefficient, *Z*_7_) are used as an input for our optical design with peak-to-valley (PV) amplitudes of (a) ±0.24, (b) ±0.49, and (c) ±0.73 *μ*m. (a)–(c) The corresponding PSF is plotted after focusing through an aspheric lens, with a Strehl ratio of (a) 0.27, (b) 0.11, (c) 0.08. (d)–(f) The corresponding PSF is plotted after focusing through an aspheric lens and optimizing the liquid–liquid interface of the electrowetting prism array with a corrected Strehl ratio of ∼0.9 for all cases. The Strehl ratio shows a significant improvement of the PSF satisfying the Maréchal criterion.

In the last example, we use the *Z*_4_ through *Z*_12_ Zernike coefficients as an input aberration consisting of defocus (*Z*_4_), two astigmatism (*Z*_5_ and *Z*_6_), two coma (*Z*_7_ and *Z*_8_), two trefoil (*Z*_9_ and *Z*_10_), spherical (*Z*_11_), and secondary astigmatism (*Z*_12_), excluding tip/tilt and piston Zernike terms. The randomly generated Zernike terms were restricted in amplitude to ±0.8 *μ*m, with values of, *Z*_4_: −0.10, *Z*_5_: −0.14, *Z*_6_: 0.13, *Z*_7_: 0.004, *Z*_8_: 0.09, *Z*_9_: 0.05, *Z*_10_: −0.02, *Z*_11_: 0.12, and *Z*_12_: 0.08 *μ*m. The Zernike polynomials coefficients are shown in Fig. 4(a). The results of adding up these Zernike terms are shown in Fig. 4(b) with peak-to-valley presented in the colored bar. Propagating a beam through with this input aberration results in a PSF with a Strehl ratio of 0.04, as shown in Fig. 4(c). Such a poor PSF is unusable for any optical system. By adjusting the curvature and tip/tilt of our electrowetting prism as shown in Fig. 1(a), we are able to optimize the Strehl ratio to 0.85 as depicted in Fig. 4(d). We have examined the limitation of our design by increasing the amplitude of the input aberrations to above ±1 *μ*m for each Zernike coefficients and we observed that the aberrated beam is obstructed by the electrowetting array at the focal plane of the fixed microlens array. This obstruction causes vignetting which results in the power loss as a consequence. Zernike coefficients with amplitude below ±1 *μ*m are fully corrected within the Maréchal criterion with no vignetting. Our simulations show that adaptive electrowetting array can be used as an alternative approach to compensate for aberrations in optical systems in the transmissive configuration.

**FIG. 4.**
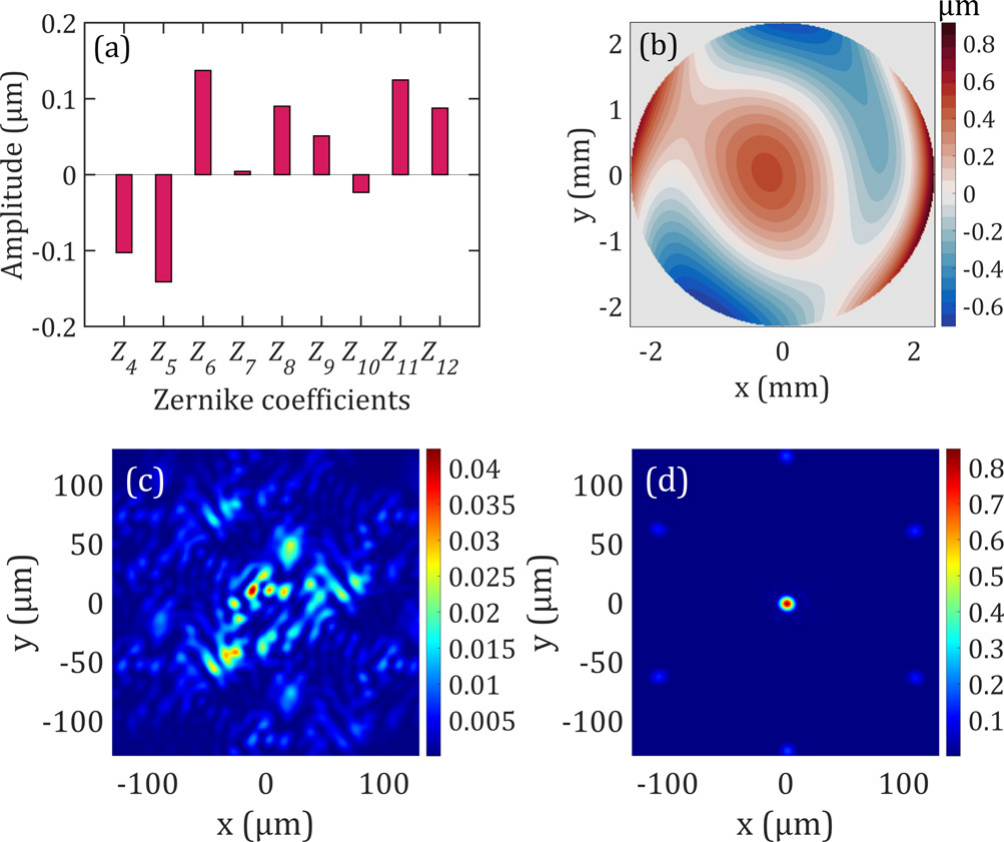
(a) Random Zernike polynomial coefficients used for wavefront aberrations and (b) the corresponding aberrated surface with peak and valley presented by the colored bar. *Z*_4_: defocus, (*Z*_5_ and *Z*_6_): astigmatism, (*Z*_7_ and *Z*_8_): coma, (*Z*_9_ and *Z*_10_): trefoil, *Z*_11_: spherical aberration, and *Z*_12_: secondary astigmatism. (c) The corresponding PSF for the given Zernike coefficients in (a) is plotted after focusing through an aspheric lens, with a Strehl ratio ∼0.04. (d) The corrected PSF is plotted after focusing through an aspheric lens, and optimizing the liquid–liquid interface of the electrowetting prism array with a Strehl ratio of 0.85. The Strehl ratio shows a significant improvement of the PSF satisfying the Maréchal criterion.

Simulations are performed to investigate the ability to correct wavefront aberrations using multielectrode electrowetting array devices. We have implemented a 127 fixed microlens array followed by an electrowetting prism array for wavefront shaping. Our optical design relies on a high-fill factor (90.7%) fixed microlens array and a lower fill factor (66.6%) electrowetting prism array. The high-fill factor fixed microlens arrays have been demonstrated using various fabrication methods, although fabricating a high-fill factor adaptive array is challenging. Hence, our approach based on a low-fill factor relaxes many fabrication constraints for an adaptive array, i.e., an electrowetting prism array in our case. To investigate the ability to correct wavefront aberrations, we have examined various aberrations such as astigmatism, coma, defocus, spherical, and trefoil with amplitudes limited to maximum range of ±1 *μ*m. The aberration amplitude range is chosen based on the reported values on various studies in eye vision and multiphoton microscopy.^13,16,22–24,102–105^ To optimize the design, we have evaluated the PSF at the focus of an aberration-free aspheric lens and have used Strehl ratio maximization based on the optical path difference approach. Our results show significant improvement of the Strehl ratio ranging from 0.85 to 0.95 after correction (starting Strehl ratio of ∼0–0.2 before correction), which satisfies the Maréchal criterion of Strehl ratio >0.8. The optical loss in our design is limited to the fill factor of the fixed microlens array which is >90.7%. In addition, the response time of electrowetting arrays is a function of their size and the choice of liquids. For instance, the switching time of an electrowetting lens is proportional to their radius,^43^ $\tau \propto r^{\left(3/2\right)}$. Our approach results in a high-speed aberration correction in the transmissive configuration with low loss. We have demonstrated the capability of implementing an electrowetting array as an adaptive optical element for aberration correction in optical systems. Multiple low and high-order aberrations are corrected using the adaptive array. Our simulations provide an effective approach implementing these arrays for aberration correction in various systems, such as free space and fiber-coupled microscopy.

## Data Availability

The data that support the findings of this study are available on request from the corresponding author. The data are not publicly available.
